# The effect of sodium-glucose cotransporter 2 inhibition mediated by blood metabolites in lymphocytic leukemia

**DOI:** 10.1016/j.gendis.2025.101664

**Published:** 2025-05-02

**Authors:** Diguang Wen, Kai Lei, Xiang Ma, Jianchuan Deng

**Affiliations:** Department of Hematology, Second Affiliated Hospital of Chongqing Medical University, Chongqing 400010, China; Hepatobiliary Surgery Department, Second Affiliated Hospital of Chongqing Medical University, Chongqing 400010, China; Hepatobiliary Surgery Department, Second Affiliated Hospital of Chongqing Medical University, Chongqing 400010, China; Department of Hematology, Second Affiliated Hospital of Chongqing Medical University, Chongqing 400010, China

Sodium-glucose cotransporter 2 (SGLT2) inhibitors are a class of oral antidiabetic drugs, but they appear to have additional metabolic effects on circulating metabolites. At present, relevant studies have also proved that SGLT2 inhibition is related to the generation of diseases through metabolites.^1^^,^^2^ However, the impact of SGLT2 inhibition on cancer still needs to be explored. This study aimed to analyze the relationship between SGLT2 inhibition, blood metabolites, and lymphocytic leukemia by Mendelian randomization analysis.

A two-sample, two-step Mendelian randomization analysis was used to explore the association between SGLT2 inhibition and lymphocytic leukemia and the mediating role of blood metabolites (Fig. 1). Genetic variants of SGLT2 inhibitors were selected via the subsequent steps, as detailed in previous studies.^3^ To start with, a tool variable representing the impact of SGLT2 inhibitors on treatment was established by employing genetic loci related to mRNA expression. Genetic variants associated with SGLT2 mRNA expression levels were chosen from the public data sets of the Genetics of Tissue Expression (GTEx) project and the eQTLGen Consortium, and single nucleotide polymorphisms within ±250 kb of the genetic loci significantly associated with the trait at the genome-wide significance level (*P* < 0.001) were filtered. Thirdly, considering the glucose-lowering effect of SGLT2 inhibitors, the correlation between each SGLT2 variant and HbA1c levels (a marker of the glucose-lowering effect) was estimated, and variants significantly associated with HbA1c (*P* < 1 × 10^−4^) were selected. The GWAS data of HbA1c originated from the UK Biobank, encompassing 344,182 individuals of European ancestry (File S1 and Table S1). Eventually, colocalization analysis between SGLT2 and HbA1c was conducted, using a posterior probability threshold of > 70% as evidence of colocalization, and clustering analysis was carried out using PLINK to eliminate single nucleotide polymorphisms with strong linkage disequilibrium (*r*^2^ = 0.8; kb = 250) based on the 1000 Genomes Project reference panel of European ancestry individuals (Table S1). Through the above steps, a total of 14 independent single nucleotide polymorphisms were selected as instrumental variables for SGLT2 inhibition (Table S2). Mendelian randomization analysis was used to assess the effect of SGLT2 inhibition on lymphocytic leukemia. Firstly, we analyzed the metabolites that influenced lymphocytic leukemia among 1400 blood metabolites (β2) using Mendelian randomization analysis and then evaluated the effect of SGLT2 inhibition on these blood metabolites (β1). The mediating proportion of each blood metabolite in the association between SGLT2 inhibition and lymphocytic leukemia was calculated as the product of β1 and β2 divided by the total effect of SGLT2 inhibition on lymphocytic leukemia.

**Figure 1:**
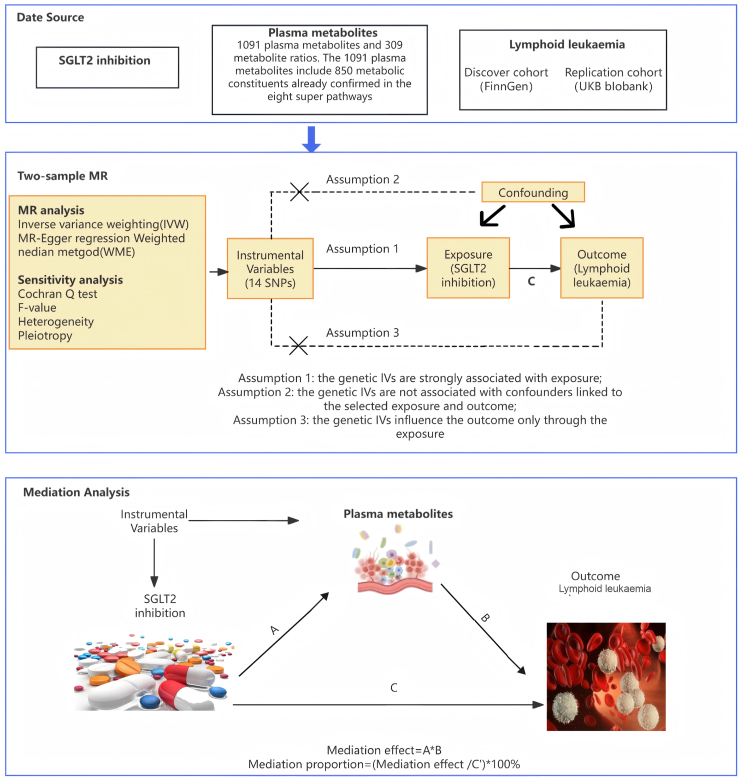
The flowchart of two-sample and two-step Mendelian randomization evaluating the effects of plasma metabolites in mediating the effect of SGLT-2 inhibition on lymphoid leukemia. HbA1c, glycated hemoglobin; GWAS, genome-wide association study; pQTL, protein quantitative trait loci; SNP, single nucleotide polymorphism; LD, linkage disequilibrium; SGLT2, sodium-glucose cotransporter 2.

In both the discovery cohort (29.53 [2.56–341.26], *P* = 6.75E-03) and the replication cohort (1.01 [1.00–1.01], *P* = 0.0318), we found that SGLT2 inhibition was associated with an increased risk of lymphocytic leukemia by Mendelian randomization analysis for every 1-SD reduction in HbA 1c (Table S3 and Fig. S1). We estimated the effect of blood metabolites on lymphocytic leukemia among 1400 metabolites using Mendelian randomization analysis and found that five metabolites were significantly associated with lymphocytic leukemia (Bonferroni corrected *P*-value threshold = 3.571E-05 [0.05/1400]) (Table S4). For urate levels, we observed a positive association with the risk of lymphocytic leukemia (odds ratio: 1.52 [95% confidence interval: 1.25, 1.86], *P* = 2.76E-05 < 3.571E-05). We further estimated the effect of SGLT2 inhibition on these five blood metabolites and observed that urate levels were significantly associated with SGLT2 inhibition (Bonferroni corrected *P*-value threshold = 0.01 [0.05/5]) (Table S5 and Fig. S2). SGLT2 inhibition was significantly associated with urate levels (odds ratio: 2.30 [95% confidence interval: 1.25, 4.25], *P* = 0.01). The proportion of SGLT2 inhibition mediated indirect effects of urate levels on lymphocytic leukemia was 10.30% (Table S6 and Fig. S3).

SGLT2 inhibitors, as widely used antidiabetic drugs, have been found to have additional metabolic effects in addition to their glucose-lowering effects. By affecting circulating metabolites to prevent the onset of heart disease, SGLT2 inhibitors have shown protective effects on organ function.^1^ At the same time, previous studies have shown that SGLT2 inhibitors also affect cancer. On the one hand, it can reduce the risk of some cancers, on the other hand, it also has the possibility of increasing some cancers.^4^ In this study, Mendelian randomization analysis was used to explore the relationship between SGLT2 inhibition, blood metabolites, and lymphocytic leukemia in the general population. Our study suggests that SGLT2 inhibitors may increase the risk of lymphocytic leukemia by affecting urate levels. Urate levels mediated the association between SGLT2 inhibition and risk for lymphocytic leukemia by approximately 10.30%. SGLT2 is a Na^+^/glucose cotransporter, and SGLT2 inhibition may lead to changes in urine composition that affect urate levels. Leukemia will also have abnormal urate levels because of abnormal purine metabolism. Abnormal urate levels are associated with leukemia and SGLT2 inhibition. This provides a potential basis for the role of urate levels in the relationship between SGLT2 inhibition and leukemia.^5^ Glucagon-like peptide-1 (GLP-1) receptor agonists do not affect patient urine composition, therefore the glucose-lowering drug of choice for patients with diabetes who are at high risk for leukemia is GLP-1 receptor agonists, which can also significantly reduce the risk of cardiovascular disease. This study provides a promising prospect for intervention in the treatment of lymphocytic leukemia.

In conclusion, this study provides genetic support for the relationship between SGLT2 inhibition, blood markers, and lymphocytic leukemia. The present study found that uric acid level seems to mediate the effect of SGLT2 inhibition on lymphocytic leukemia. However, the genetic data used in this study came from a European population, so the conclusions may have ethnic differences and need to be verified in different populations.

## CRediT authorship contribution statement

**Diguang Wen:** Writing – original draft, Project administration, Data curation. **Kai Lei:** Writing – original draft, Supervision, Software. **Xiang Ma:** Software, Funding acquisition. **Jianchuan Deng:** Writing – original draft, Investigation, Funding acquisition.

## Data availability

All data of the article can be obtained from the corresponding author with reasonable request.

## Funding

This project was supported by the Key Project of Chongqing Natural Science Foundation of China (No. CSTB2023NSCQ-LZX0052).

## Conflict of interests

No benefits in any form have been received or will be received from a commercial party related directly or indirectly to the subject of this article.

## References

[1] Heerspink H.J., Perkins B.A., Fitchett D.H., Husain M., Cherney D.Z. (2016). Sodium glucose cotransporter 2 inhibitors in the treatment of diabetes mellitus: cardiovascular and kidney effects, potential mechanisms, and clinical applications. Circulation.

[2] Xu Y., Zhang C., Jiang K. (2023). Structural repurposing of SGLT2 inhibitor empagliflozin for strengthening anti-heart failure activity with lower glycosuria. Acta Pharm Sin B.

[3] Li J., Yu Y., Sun Y. (2023). SGLT2 inhibition, circulating metabolites, and atrial fibrillation: a Mendelian randomization study. Cardiovasc Diabetol.

[4] Nakachi S., Okamoto S., Tamaki K. (2022). Impact of anti-diabetic sodium-glucose cotransporter 2 inhibitors on tumor growth of intractable hematological malignancy in humans. Biomed Pharmacother.

[5] Sridhar V.S., Cosentino F., Dagogo-Jack S. (2024). Effects of ertugliflozin on uric acid and gout-related outcomes in persons with type 2 diabetes and cardiovascular disease: post hoc analyses from VERTIS CV. Diabetes Obes Metabol.

