# Supplementary material for: The effect of sodium-glucose cotransporter 2 inhibition mediated by blood metabolites in lymphocytic leukemia
Source: Genes Dis. 2025 May 2;12(6):101664. doi: 10.1016/j.gendis.2025.101664 (PMC12270927; doi:10.1016/j.gendis.2025.101664)
Supplement: Multimedia component 1 [file mmc1.docx]

**Introduction to the Supplementary material (Title: The Effect of Sodium‑glucose Cotransporter 2 Inhibition Mediated by Blood Metabolites in Lymphocytic Leukemia)**

The supplementary materials of this study, including supplementary tables and pictures, are annotated. The supplementary materials of this study mainly show the research process and data statistics involved in the study. An instrumental variable of SGLT2 inhibition was identified, and a two-sample Mendelian randomization study was conducted to confirm its association with leukemia. We then conducted a two-sample Mendelian randomization study to identify the potential metabolites between SGLT2 inhibition and lymphocytic leukemia, and finally calculated the proportion of metabolites mediating the relationship.
