# Supplementary material for: The effect of sodium-glucose cotransporter 2 inhibition mediated by blood metabolites in lymphocytic leukemia
Source: Genes Dis. 2025 May 2;12(6):101664. doi: 10.1016/j.gendis.2025.101664 (PMC12270927; doi:10.1016/j.gendis.2025.101664)
Supplement: Multimedia component 2 [file mmc2.docx]

**Supplementary Methods**

**Study Design**

The overall study design is illustrated in Figure 1. Initially, we obtained the instrumental variables (IVs) for SGLT2 inhibitors as previously reported and conducted a two-sample Mendelian randomization (MR) analysis to evaluate the causal relationship among SGLT2 inhibition, blood metabolites, and lymphoid leukemia. Subsequently, a two-step MR approach was employed to perform mediation analysis and to calculate the proportion of mediation.

**Instrumental Variables for SGLT2 Inhibition**

Instrumental variables for SGLT2 inhibition were selected using summary statistics from the UK Biobank, comprising 344,182 non-diabetic individuals of European ancestry, following previously reported methodologies [1,2]. Briefly, drug targets of SGLT2 inhibitors and their corresponding coding genes were identified. Genetic variants significantly associated with blood glucose biomarker levels (HbA1c) were then selected as IVs.

**Metabolite GWAS Data Sources and Instrumental Variable Selection**

Genome-wide association study (GWAS) summary statistics for 1400 blood metabolites were obtained from the GWAS Catalog (GCST90199621-GCST90201020). This dataset includes 1091 metabolites and 309 metabolite ratios from 8299 individuals enrolled in the Canadian Longitudinal Study on Aging (CLSA) cohort [3,4]. IVs for metabolites were selected based on the following criteria: a significance threshold of P < 1 × 10−5, linkage disequilibrium (LD) threshold of r2 < 0.1, and a clumping distance of 500 kb [5].

**Leukemia GWAS Data Sources**

To reliably infer causal relationships, GWAS summary data for lymphoid leukemia were obtained from two large-scale biobanks, comprising the discovery and validation cohorts. Specifically, the discovery cohort included data from the Finnish Biobank (GWAS ID: "finn-b-CD2_LYMPHOID_LEUKAEMIA"), and the validation cohort utilized data from the UK Biobank (GWAS ID: "ieu-b-4956"). Data access was facilitated via the MR-Base platform (https://gwas.mrcieu.ac.uk/) using the TwoSampleMR R package (v0.6.8) [6].

**Statistical Analysis**

Two-sample MR analysis was performed to evaluate the causal effect of SGLT2 inhibition on lymphoid leukemia risk. The inverse variance weighted (IVW) method served as the primary analytical approach. To ensure the robustness of the findings, additional MR methods were employed, including MR-Egger regression, weighted median, simple mode, and weighted mode approaches. For analyses where only a single IV was available, the Wald ratio method was applied.

When a positive causal relationship among SGLT2 inhibition, specific metabolites, and lymphoid leukemia was observed, a two-step MR mediation analysis was conducted to explore whether circulating metabolites mediated the causal association. Specifically, we first estimated the effect of SGLT2 inhibition on circulating metabolite levels using MR, followed by an assessment of the causal impact of these metabolites on lymphoid leukemia. Mediation analyses were performed for metabolites that demonstrated significant associations with both SGLT2 inhibition and leukemia risk. The proportion of mediation was quantified by decomposing the "total effect" into "direct" and "indirect" effects. Standard errors (SE) and confidence intervals (CI) for mediation proportions were estimated using the Delta method [7].

References

1. Li, J., et al. SGLT2 inhibition, circulating metabolites, and atrial fibrillation: a Mendelian randomization study. Cardiovasc Diabetol, 2023, 22(1): 278. https://doi.org/10.1186/s12933-023-02019-8

2. Zhao, S.S., et al. Sodium-glucose cotransporter 1 inhibition and gout: Mendelian randomisation study. Semin Arthritis Rheum, 2022, 56: 152058. https://doi.org/10.1016/j.semarthrit.2022.152058

3. Chen, Y., et al. Genomic atlas of the plasma metabolome prioritizes metabolites implicated in human diseases. Nat Genet, 2023, 55(1): 44-53. https://doi.org/10.1038/s41588-022-01270-1

4. Kettunen, J., et al. Genome-wide association study identifies multiple loci influencing human serum metabolite levels. Nat Genet, 2012, 44(3): 269-276. https://doi.org/10.1038/ng.1073

5. Bulik-Sullivan, B.K., et al. LD Score regression distinguishes confounding from polygenicity in genome-wide association studies. Nat Genet, 2015, 47(3): 291-295. https://doi.org/10.1038/ng.3211

6. Hemani, G., et al. The MR-Base platform supports systematic causal inference across the human phenome. eLife, 2018, 7: e34408. https://doi.org/10.7554/eLife.34408

7. Carter, A.R., et al. Mendelian randomisation for mediation analysis: current methods and challenges for implementation. Eur J Epidemiol, 2021, 36(5): 465-478. https://doi.org/10.1007/s10654-021-00757-1
