# Supplementary material for: The effect of sodium-glucose cotransporter 2 inhibition mediated by blood metabolites in lymphocytic leukemia
Source: Genes Dis. 2025 May 2;12(6):101664. doi: 10.1016/j.gendis.2025.101664 (PMC12270927; doi:10.1016/j.gendis.2025.101664)
Supplement: Multimedia component 4 [file mmc4.pdf]

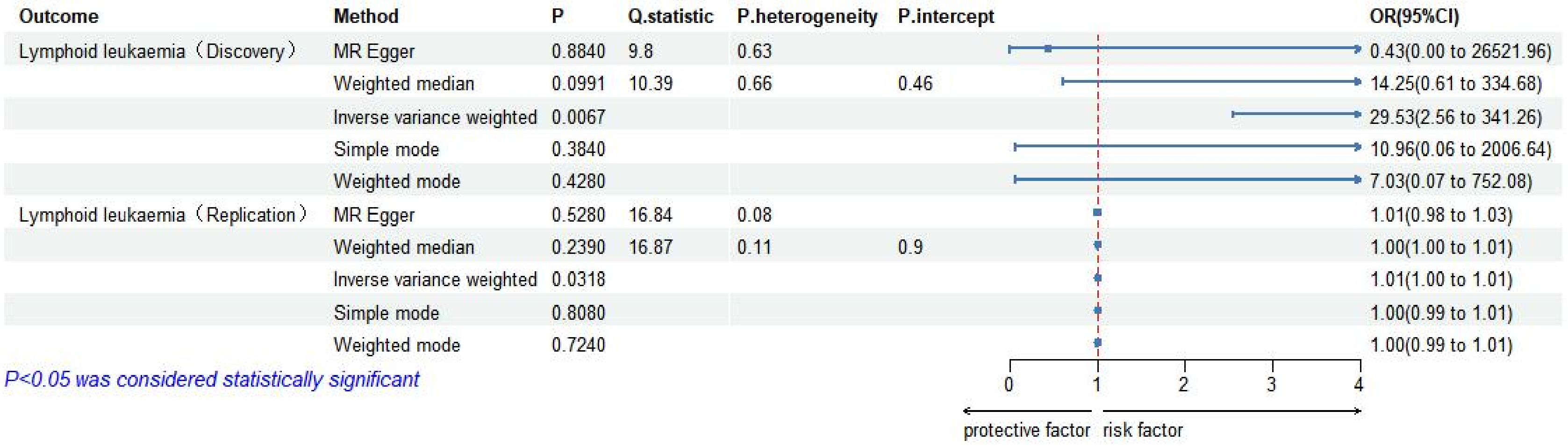

Supplementary Figure 1. **The forest plot of showing the effects of SGLT-2 inhibition on lymphoid leukaemia in both the Discovery cohort and the Replication cohort. OR, Odds Ratio; CI, Confidence interval; SGLT2, Sodium-glucose cotransporter 2.**

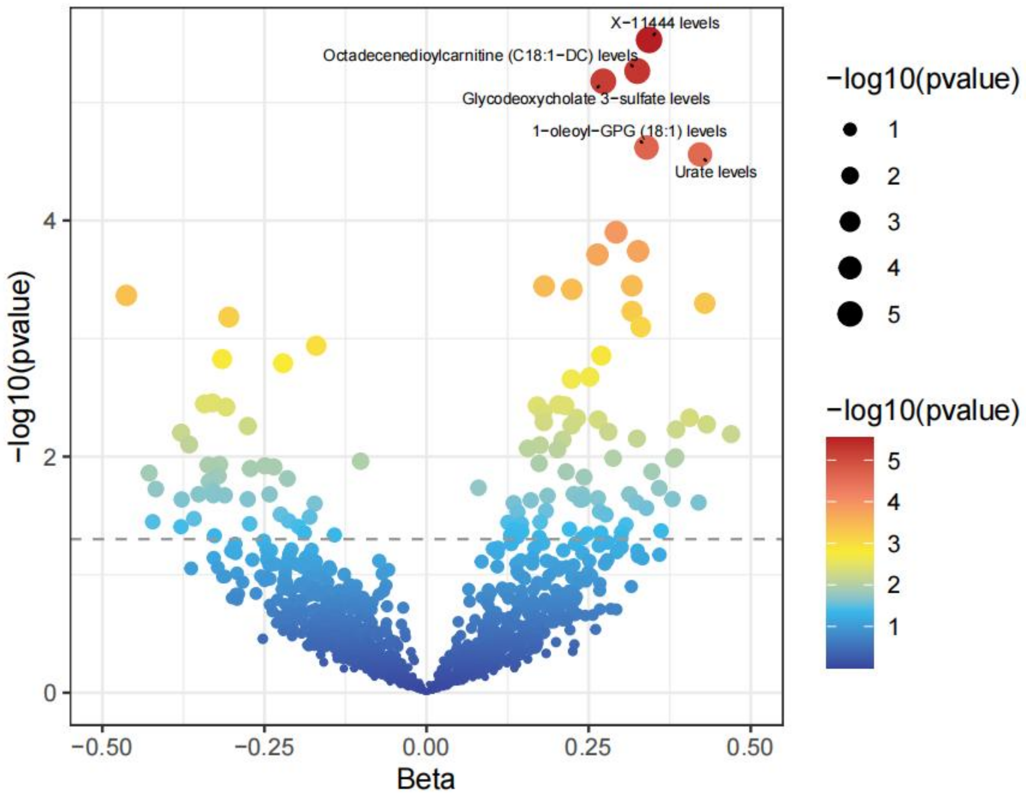

Supplementary Figure 2. **The Volcano plots between positive metabolites and outcomes.**

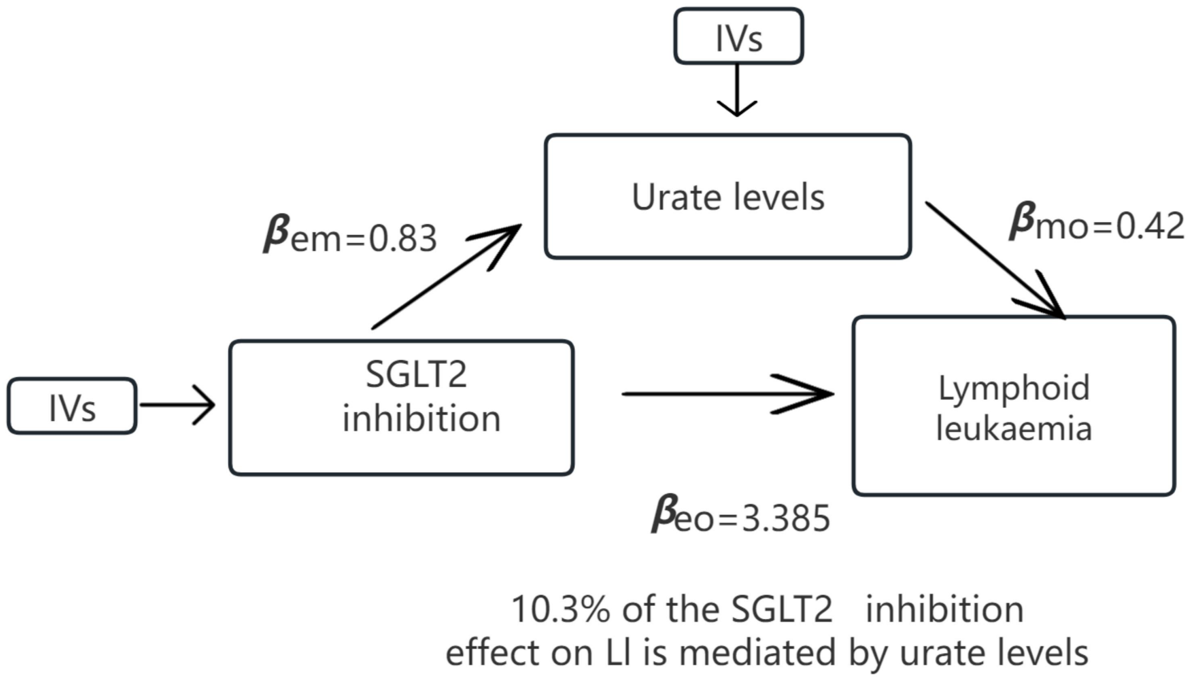

Supplementary Figure 3. **The Urate levels mediated the causal effect of SGLT2 inhibitor on lymphoid leukaemia. IV, Instrumental variable; SGLT2, Sodium-glucose cotransporter 2.**
